# Epidemiology and future risk estimates of cutaneous leishmaniasis in district Dera Ismail Khan, Pakistan: analysis of data from 2019-2022

**DOI:** 10.4314/ahs.v24i4.8

**Published:** 2024-12

**Authors:** Aqsa Mansoor, Kiran Afshan, Ghulam Narjis, Sabika Firasat

**Affiliations:** 1 Department of Zoology, Faculty of Biological Sciences, Quaid-i-Azam University, Islamabad, 45320, Pakistan; 2 Department of Statistics, Rawalpindi Women University, 6^th^ Road, Satellite Town, Rawalpindi, Punjab, Pakistan

**Keywords:** Cutaneous leishmaniasis, spatiotemporal analysis, Pakistan

## Abstract

**Background:**

Cutaneous leishmaniasis (CL), despite not being a life-threatening condition, has a devastating impact on the public health. CL is widely distributed, exhibiting a distinct epidemiological pattern all over the world. The aim of this study was to investigate CL in District Dera Ismail Khan, Khyber Pakhtunkhwa, Pakistan, and to estimate the risk of epidemics.

**Objectives:**

**Materials and Methods:**

From 2019 to 2022, 1135 CL patients' epidemiological data were collected from district health facilities. For epidemiological characterization, descriptive statistics were used. The spatial analysis was done using ArcGIS V.10.3. The relationship between CL occurrence and climatic variables was investigated using liner regression analysis.

**Results:**

Between 2019 and 2022, there was a decline in the annual CL incidence trend. Males and people under the age of 20 were particularly susceptible. A total of 1204 lesions were identified, with 76.1% of individuals having a single lesion and 23.9% having multiple lesions. Most of the lesions were nodular and ulcerative in nature and were found on exposed body parts such as the lower extremity (34.2%) and the face (30.7%). A choropleth map revealed an increased incidence of CL in Tehsil D.I.K (63%) and Paharpur (10%). According to a digital elevation model, high altitudes have a lower prevalence of CL. For focal transmission and high-risk zones, Inverse Density Weight (IDW) spatial interplation, focal statistics, cluster, and outlier analysis validated that CL cases were high in D.I.K, Kulachi, and Paharpur tehsils. Increased temperature, relative humidity, and precipitation were not significantly associated with CL infection.

**Conclusions:**

The study provided essential details for public health sectors to develop intervention strategies for future CL outbreaks.

## Introduction

Leishmaniasis is a vector-borne disease caused by obligate intracellular protozoan parasites of the genus Leishmania and transmitted by female sandfly bites of the Phlebotomus and Lutzomyia species^1^. Leishmaniasis has three clinical manifestations: visceral leishmaniasis (VL), mucocutaneous leishmaniasis (MCL), and cutaneous leishmaniasis (CL). Unlike visceral and mucocutaneous leishmaniasis, cutaneous leishmaniasis is not fatal 2. According to the World Health Organisation (WHO), this disease exists in 98 countries, with an estimated 20,000-40,000 deaths and 12 million cases at risk per year as of 2020. The yearly prevalence of new cases of CL and VL ranges from 0.7-1.2 million and 0.2-0.4 million cases, respectively^3^. It is one of the world's most overlooked poverty-related illnesses, affecting the most vulnerable populations in developing nations, and is linked to migration, malnutrition, immune system deficiencies, insufficient healthcare services, inadequate schooling, illiteracy, and disparitis in gender^4^.

CL infection is more common in developing countries, particularly Afghanistan, Iraq, Iran, Peru, Syria, Brazil, Algeria, and Pakistan 5,6. CL had a significant impact on people in Pakistan, particularly in Khyber Pakhtunkhwa (KP) province, and several studies reported the disease in southern KP 7,8. A 2.7% prevalence rate has been noted in the country's NorthWest 9, and 37 species of sand flies are responsible for spreading the illness to healthy hosts^10^. The most recent epidemic resulted in nearly 28,000 cases of leishmaniasis in KP province^11^. The infection, caused by *L. tropica* and *L. major*, is primarily spreading in Khyber Pakhtunkhwa because of the immigration of several million refugees^12^. Numerous endemic foci have been regularly identified in Khyber Pakhtunkhwa's settled districts, such as Dera Ismail Khan^13^, Kohat^7^, Karak^14^, Dir^15^, Sawat^16^, Charsadda^17^, and Malakand^18^, and among others from various tribal agencies^12^, ^19^.

The province of Khyber Pakhtunkhwa is in northwest Pakistan and shares a nearly 2300 km long border (Durand line) with Afghanistan. The capital of Afghanistan, Kabul, has a population of 3.7 million people and is located 290 kilometres from Peshawar (KP), which is believed to have the highest incidence of CL in the world^20^. With a dry, hot, and humid climate, an abundance of sand-fly vectors, and cross-border movements, the prevalence of CL is high in the KP and Federally Administered Tribal Area FATA along the Pakistan-Afghan border 20. Few studies in KP investigated the Geographic Information Systems (GIS)-based consideration on leishmaniasis^15^, ^21^, while new CL epidemics have been detected in previously unexplored endemic regions 12. The efficiency of control measures, however, has been hampered by a lack of disease surveillance and vector data. As a result, the purpose of this study was to provide epidemiological status and risk maps for CL in the Dera Ismail Khan Districts from 2019 to 2022. ArcGIS software was used to analyze the spatial distribution of CL to investigate the future epidemic risk.

## Materials And Methods

### Study Area

**Figure 1:**
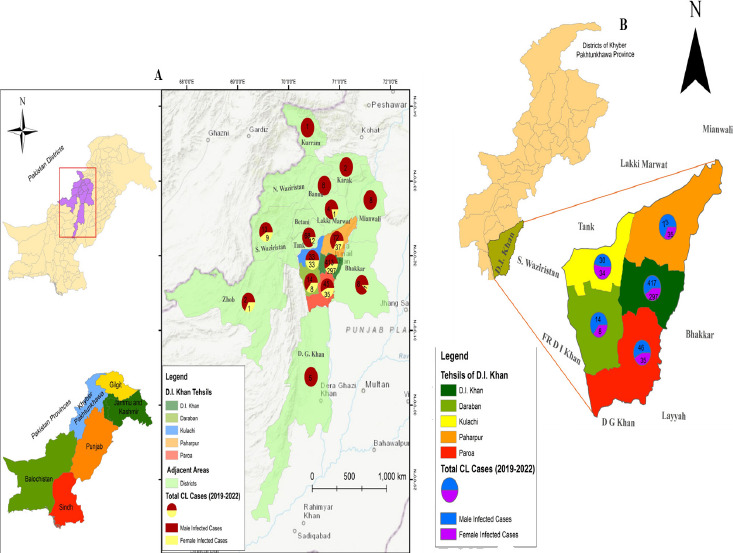
Map of study area indicating location of sampled union councils with defined male and female ratio (A) D.I. Khan and adjoining areas (B) D.I Khan district

The current study was conducted in Dera Ismail Khan, which is in the province KP and has latitude 31.8626° N and longitude 70.9019° E. The study area has a dry, dusty environment with warm summers and mild winters and is thought to be one of the most significant endemic locations for CL. The district is made up of five tehsils: D.I.K, Daraban, Kulachi, Paharpur, and Paroa. The data also includes the number of patients in neighbouring areas named Tank, D.G. Khan, Lakki marwat, Mianwali, Bhakkar, Waziristan, Bannu, and Karak, among others (Fig.1).

### Ethical consideration and consent

The current study was conducted using secondary data that did not include patient names or personal information. The study was approved by the Office of the District Health Officer, Dera Ismail Khan Khyber Pakhtunkhwa Pakistan with Ref. No: 7799-7801.

### Collection of Leishmaniasis health record

Tehsil-wise epidemiological data of microscopically declared CL positive patients were collected from various local hospitals and CL centers, including the district Headquarter Hospital D.I. Khan and the Tehsil Headquarter Hospital Kohat. The research was carried out between January 2019 and March 2022. Patients from different sexes, socioeconomic levels, and ages are included in the study. For descriptive analysis, the obtained data were entered into Microsoft Excel. Google Earth Pro (version 7.3) coordinates were used to get the districts' locations, and the average incidence of CL in each district was aligned accordingly. Spatial risk and statistical analysis were conducted using ArcGIS (version 10.7.1). The proportional symbolized map and extracted Digital Elevation Model (DEM) (data from http://www.diva-gis.org/datadown) were also used to determine the spatial risk of CL infection, and Zonal Statistics was applied to the DEM raster output data to determine the precise elevation of each infected tehsil.

### Spatial Analysis

By displaying the quantity of CL cases with continuous colours in a lack of district fidelity, a choropleth map was created. Field statistics were used to confirm the model's accuracy^22^. To assess CL transmission, spatial interpolation was done using the Inverse Distance Weight (IDW) method. IDW is an estimation technique that uses a linear combination of known sampling point values to forecast unknown non-sampling point values with corresponding weighted values of inverse distance^23^, ^24^. The calculation was performed with the settings of neighbouring type, 0.5 smoothing factor, and zero spatial pattern angles.

The ArcGIS spatial statistics toolkit was employed. For each significant feature, a local Moran's I value, a z-score, a pseudo p-value, and a code designating the cluster type were calculated (Anselin Local Moran's I). To ensure that every feature had at least one neighbour, a fixed distance band was used. The neighbouring zone around the indices was measured using the Euclidean distance method. According to Pakzad et al.^22^, spatial weights were not uniformly distributed.

### Statistical analysis

The collected epidemiological data were entered into Microsoft Excel, and statistical analysis was performed using SPSS version 21 (IBM Corp., Armonk, NY). To identify statistically significant differences between independent variables, one-way ANNOVA was used. The climatic data along with CL incidence was evaluated with liner regression. The 95% Confidence Interval (CI) and a significant P-value of 0.05 were considered for all statistical analysis.

## Results

A total of 1135 microscopically confirmed CL positive cases have been reported in the Dera Ismail Khan District over the course of the previous four years. From 2019 (n=423, 37.3%) to 2022 (n=78, 6.9%), CL incidence dropped significantly. CL cases were more common in the D.I.K tehsil (n= 712, 62.7%) and adjacent areas of the district (n=146, 12.6%) then Paharpur (n=109, 9.6%), Paroa (n=80, 7%), Kulachi (n=66, 5.8%) and Daraban (n=22, 1.95). The difference between CL prevalence between tehsil was statistically significant (R=, 0.57, P=0.001). Gender-based annual distribution of CL cases showed a similar pattern (R=0.194, P= 0.316) of infection in both males (n=700, 61.7%) and females (n=435, 38.3%). The 1-20- year age group (n=734, 64.7%) had significantly (R= 0.556, P= 0.005) the highest annual incidence of CL, then old age groups. The epidemiological findings showed a variety of CL infection patterns. Cutaneous lesions, ulcers, and nodules have been identified as the most frequent clinical characteristics. A total of 1204 lesions were recorded among the 1135 patients. Cutaneous lesions occurrence was from single lesion (n=864, 76.1%) to multiple lesions (n=271, 23.9%) (R= 0.329, P=0.08). Most of the lesions were seen on exposed body areas mainly on face (n=348, 30.7%) followed by upper extremity (n=330, 29.1%), lower extremity (n=388, 34.2%) and mixed (n=69, 6.1%). However, site of lesions did not vary significantly (R= 0.194,; P=0.9) with the study areas. The interval between the beginning of the CL lesion and the diagnosis might range from less than two months to more than a year. The duration of lesion was 1-2 months in most of the CL patients (n=648, 57.1%) followed by 3-4 months (n=272, 24%), 4-5 months (n=83, 7.3%), 7-8 months (n=89, 7.8%) and greater than 9 months (n=43, 3.8%) in study period. The result showed statistically significant (R=0.503, P=0.005) reduction in number of lesions with an increase in months (Table 1).

**Table 1:** Demographic and clinico-epidemiological characteristics of the studied population (2019-2022)

Variables		2019 n (%)	2020 n (%)	2021 n (%)	2022 n (%)	Total (%)	R(adjusted)	R(squared)	P-value
**Annual Incidence**		423(37.3)	419 (36.9)	215(18.9)	78(6.9)	1135 (100)			
**Age (years)**	1-20	262(70)	271(65)	146(68)	55(71)	734(64.7)			
	21-40	114(27)	95(23)	46(21.3)	19(24.3)	274(24.1)	0.556	0.645	0.005^*^
	41-60	38(9)	41(10)	66(31)	2(3)	101(8.9)			
	=61	9(2.1)	12(3)	3(1.3)	2(3)	26(2.3)			
**Gender**	Male	256(61)	259(62)	132(61.3)	53(68)	700(61.7)	0.27	0.166	0.316^NS^
	Female	167(39.4)	160(38.1)	83(39)	25(32)	435(38.3)			
**No. of lesions**	Single	316(75)	341(81.3)	154(72)	53(68)	864(76.1)	0.329	0.425	0.08^NS^
	Multiple	107(25.2)	78(19)	61(28.3)	25(32)	271(23.9)			
**Site of lesions**	Face	126(30)	128(31)	69(32)	25(32)	348(30.7)			
	Upper extremity	116(27.4)	107(26)	69(32)	38(49)	330(29.1)	0.194	0.23	0.9^NS^
	Lower extremity	142(34)	154(37)	77(36)	15(19.2)	388(34.2)			
	Mixed	39(9.2)	30(7.1)	0(0)	0(0)	69(6.1)			
**Duration of lesion (months)**	1-2	239(57)	267(64)	106(49.3)	36(46.1)	648(57.1)			
	3-4	100(24)	93(22.1)	56(26)	23(29.4)	272(24)			
	5-6	31(7.3)	15(4)	25(12)	13(17)	83(7.3)	0.503	0.68	0.005^*^
	7-8	38(9)	29(7)	16(7.4)	6(8)	89(7.8)			
	=9	15(4)	15(4)	13(6)	0(0)	43(3.8)			
**Area**	D.I.K	286(68)	256(61)	129(60)	41(53)	712(62.7)			
	Daraban	6(1.4)	15(4)	1(0.4)	0(0)	22(1.9)			
	Kulachi	13(3)	43(10.2)	8(4)	2(3)	66(5.8)	0.57	0.663	0.001^**^
	Paharpur	26(6.1)	29(7)	40(19)	14(18)	109(9.6)			
	Paroa	26(6.1)	23(5.4)	19(9)	12(15.3)	80(7)			
	Adjacent areas	66(16)	53(13)	18(8.3)	9(12)	146(12.9)			

* Significant p≤0.05

** Significant p≤0.001

NS Not significant p≥0.05

**Figure 2:**
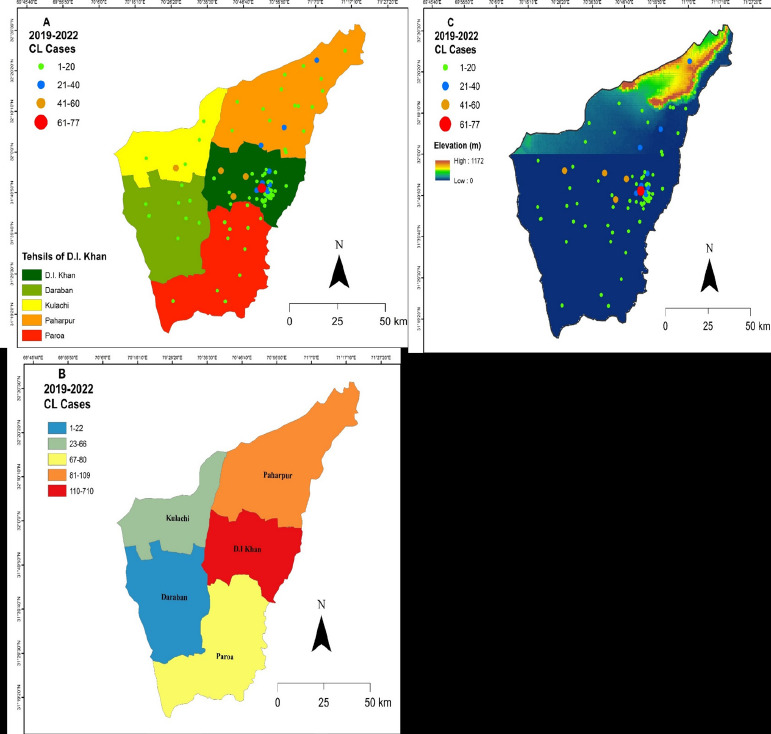
(A) Cutaneous leishmaniasis (CL) cases in each tehsil, showing the highest and lowest incidences across the district; (B) a choropleth map showing district-based CL cases; (C) a digital elevation model (DEM) map illustrating the occurrence of CL at different altitudes

The highest and least occurrence of average incidence of CL in each union council of five tehsils of Dera Ismail Khan is visually described in Figure 2A. The highest CL incidence was seen in tehsil D.I.K (n= 712, 62.7%) then Paharpur (n=109, 9.6%), Paroa (n=80, 7%) Kulachi (n=66, 5.8%), and Daraban (n=22, 1.95), according to a choropleth map (Fig. 2B). The projected CL average incidence on the extracted DEM map revealed the presence of CL cases throughout the district Dera Ismail Khan, ranging from 0 to1172m. The incidence of CL decreased with high altitudes and was found highest in Zaffar Abad and Chahkan village of tehsil D.I.K (Fig. 2C). Each village's geographical coordinates were thoroughly examined, and the highest CL incidences within a range of 0 to 178 m elevations were specified (Table 2).

**Table 2:** CL Zonal Statistics with elevation (m) of tehsils and high-risk union councils (UC) across the study period (2019-2022)

Tehsil	UC	Elevation(m)	2019 n	2020 n	2021 n	2022 n	Total cases	R(adjusted)	R(squared)	P-Value
**D.I.Khan**	AbdulKhel	195	4	0	0	0	4	**0.47**	**0.61**	0.001^**^
	AlMuizMill	186	1	0	0	0	1			
	AnjumAbad	160	0	1	1	0	2			
	Arrah	0	3	0	0	0	3			
	Bannu	178	6	2	0	0	8			
	BarkiTown	0	1	1	0	0	2			
	BastiDirkhan	166	1	2	3	1	7			
	BastiSherPao	176	1	0	0	0	1			
	BastiUstrana	178	3	1	0	0	4			
	BayPass	182	6	3	0	0	9			
	Bhakkar	190	5	2	2	0	9			
	Budhani	215	0	1	0	0	1			
	Cantt	210	0	1	0	0	1			
	Chahkan	178	17	22	14	3	60			
	Chandna	179	0	1	0	0	1			
	CRBC chowk	0	3	12	0	0	15			
	DarabanChungi	174	2	1	0	0	3			
	Darazinda	175	0	0	2	0	2			
	Derajaat	172	1	11	10	0	22			
	Dewala	178	1	9	2	2	14			
	Dinpur	0	0	2	0	0	2			
	DiyalRoad	176	5	1	0	0	6			
	FatehMorh	252	10	10	9	0	29			
	FatehPoor	171	2	0	0	0	2			
	GaraAlamKhan	233	0	1	0	0	1			
								
	GaraJamal	172	0	0	1	0	1			
	GaraMirAlam	175	0	1	0	0	1			
	GilaniTown	0	0	0	1	0	1			
	Giloti	175	0	1	0	0	1			
	Girsaal	0	1	0	0	1	2			
	Gomal	185	6	1	1	0	8			
	GreenTown	177	3	3	0	0	6			
	GridRoad	0	1	0	0	0	1			
	GulshanNisarTown	175	2	4	4	2	12			
	HajiMora	176	0	0	5	0	5			
	Hassam	175	0	1	1	0	1			
	Himmat	181	0	2	0	0	2			
	IjazAbad	175	0	1	0	0	1			
	IqbalQilla	180	14	15	0	0	29			
	KaloKaland	177	0	1	0	0	1			
	KamraniChowk	173	0	1	0	0	1			
	Kech	178	8	4	4	6	22			
	Khanii	0	5	0	0	0	5			
	Khutti	174	3	0	0	0	3			
	KiriShamozai	0	1	0	0	0	1			
	KirriAlazai	0	10	6	0	0	16			
	Kokar	200	1	0	0	0	1			
	Korai	207	11	21	10	5	47			
	KotHabib	214	0	1	0	0	1			
	KotJai	0	0	1	0	0	1			
	KotlaSaidan	0	6	5	13	3	27			
	Lachhra	209	3	15	4	6	28			
	Lok	0	1	1	0	0	2			
	MadinaColony	182	4	0	0	0	4			
	Mandhra	184	0	3	4	0	7			
	MohallaSariban	0	3	0	0	0	3			
	MolvianWaliBasti	179	1	0	0	0	1			
	Mullazai	0	4	2	0	0	6			
	MuneezAbad	0	4	0	0	0	4			
	MuqimShah	529	0	1	0	0	1			
	Muriyali	184	4	11	4	2	21			
	MusaZaiSharif	0	0	3	0	0	3			
	Nasir bagh	174	1	0	0	0	1			
	Nawab	0	0	1	0	0	1			
	NiaziChowak	0	7	0	0	0	7			
	NizamPump	183	0	4	0	0	4			
	Paroa	184	10	5	0	0	15			
	Police line	178	1	7	5	5	18			
	Pota	236	0	1	0	0	1			
	QasooriaTown	0	3	0	0	0	3			
	QureshiMorh	175	6	2	0	0	8			
	RattaKulachi	0	2	8	3	2	17			
	RehmanAbad	0	1	0	0	0	1			
	SaddarPoliceStation	0	8	2	0	0	10			
	SaidAbad	0	1	5	5	1	12			
	SaiduWala	524	2	3	2	8	15			
	ShahAlamAbad	0	1	0	0	0	1			
	ShahAlamBurki	0	0	1	0	0	1			
	Shalimar	0	0	1	0	0	1			
	SheikhAbad	173	0	1	0	0	1			
	SheikhYousif	0	1	3	0	0	4			
	ShorKot	182	7	3	2	5	17			
	Siyal	0	4	2	0	0	6			
	WandaKarimShah	0	2	0	0	0	2			
	Yarik	187	0	2	0	0	2			
	ZaffarAbad	0	58	19	0	0	77			
	Zarkani	0	0	1	0	0	1			
			
**Paharpur**	AwanAbad	180	4	0	3	0	7	**0.08**	**0.23**	**0.09^NS^**
	BandKorai	183	1	5	3	3	12			
	BilotSharif	183	2	2	0	0	4			
	DhapShumali	162	0	1	0	0	1			
	GandiUmarKhan	168	0	1	9	0	10			
	KachiMaliKhel	167	1	0	0	0	1			
	KatGarh	173	1	0	0	0	1			
	KattaKhel	175	0	4	0	0	4			
	KirriKhaisore	178	5	4	1	1	11			
	Lar	369	0	1	0	0	1			
	PaharpurTown	161	3	10	8	0	21			
	Paniala	185	3	6	0	0	9			
	Rangpur	181	0	1	2	0	3			
	RehmaniKhel	446	5	0	12	0	17			
	WandaKhanMohammad	204	0	0	5	2	7			

**Paroa**	DarabanKhurd	236	9	1	0	0	10	**0.13**	**0.22**	**0.33^NS^**
	LundaSharif	196	2	4	1	1	8			
	Mahra	228	3	4	0	1	8			
	Malana	205	0	1	1	0	2			
**Paroa**	DarabanKhurd	236	9	1	0	0	10	**0.13**	**0.22**	**0.33^NS^**
	LundaSharif	196	2	4	1	1	8			
	Mahra	228	3	4	0	1	8			
	Malana	205	0	1	1	0	2			
	Miran	249	0	0	2	6	8			
	Faqir abad	196	0	1	0	0	1			
	Naivela	178	1	4	3	3	11			
	Ramak	228	2	0	0	0	2			
	Zandani	185	2	17	1	10	30			

**Kulachi**	GaraHayat	183	3	7	2	0	12	**0.05**	**0.36**	**0.25^NS^**
	
	Hathala	217	0	2	2	0	4			
	KotWaliDad	235	1	1	0	0	2			
	Kulachi	221	10	10	10	4	34			
	Looni	212	4	2	1	0	7			
	Maddi	182	2	0	1	0	3			
	Rorri	754	1	1	0	0	2			
	WaliKot	175	1	1	0	0	2			

**Daraban**	DarabanKalan	193	6	1	0	0	7	**0.86**	**0.91**	0.04^*^
	GaraEsaKhan	188	0	0	1	0	1			
	KotEssaKhan	769	0	3	0	0	3			
	Saggu	183	8	3	0	0	11			

* Significant p≤0.05

** Significant p≤0.001

NS Not significant p≥0.05

**Figure 3:**
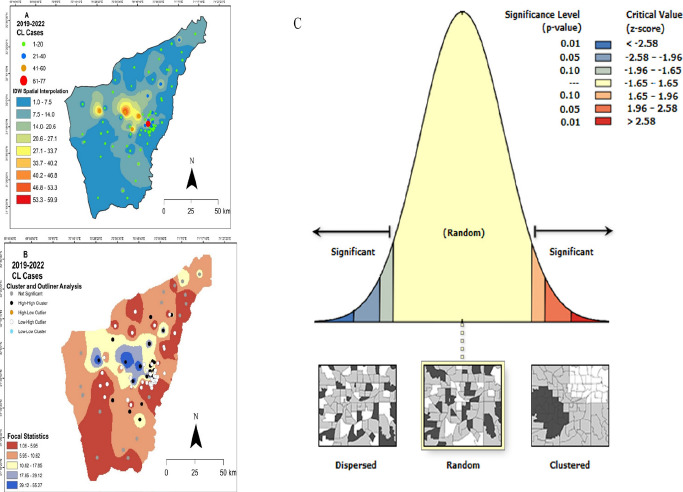
(A) Cutaneous leishmaniasis (CL) transmission analyzed by inverse distance weighting (IDW) interpolation; (B) focal statistics with cluster and outlier analysis (C) showing dispersed, random and clustered areas with significant level and critical values

**Figure 4:**
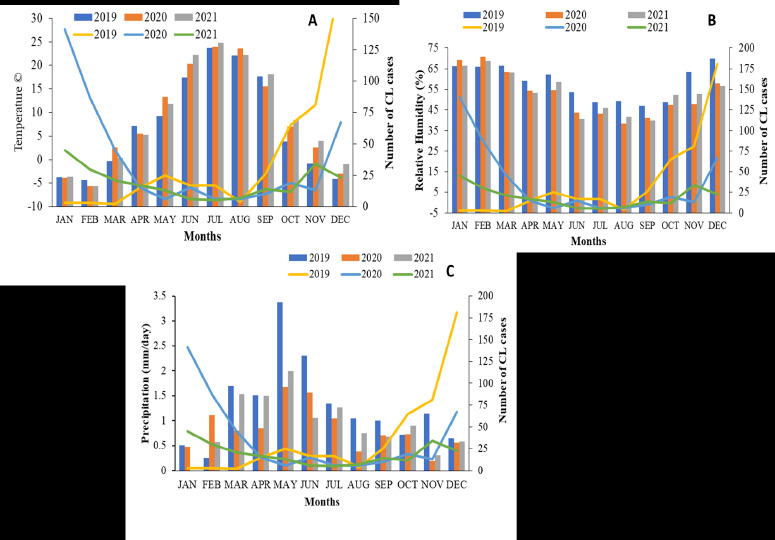
Number of CL cases association with mean monthly (A) temperature (°C) (B) relative humidity (%) (C) and precipitation (mm/day) from 2019 to 2021 of D.I.K district of KP, Pakistan

The future epidemic threats of CL infection were validated by IDW analysis, where the CL cases were high at D.I.K, Kulachi and Paharpur. The map showed CL low and high endemic areas, the focal distances were calculated using the mean values of the IDW analysis after careful observation of the transmission patterns (Fig 3A). The IDW analysis justified the threats of CL infection in the areas located closer to the highest CL presenting villages. Cluster and Outliers analysis was performed, and high cluster villages were D.I.K (z-score= 1.8, P=0.07) and Paharpur (z-score= 1.4, P=0.1). However, Kulachi (z-score= 1.0, P=0.27), Daraban (z-score= 1.4, P=0.15) and Paroa (z-score= 0.9, P=0.31) were the high and low outlier villages. The other villages were found not to be significant (Fig. 3B). The climatic data did not show significant association with CL cases in the D.I. Khan district and infection was most prevalent during the winter (Fig. 4ABC).

## Discussion

In Pakistan, the spread of CL follows intricate patterns and is ongoing throughout the surrounding areas. Several outbreaks have recently been reported in the northern regions, including Gilgit, Kashmir, and southern KP. It is believed that disease-free areas close to the boundaries of these endemic regions are also at risk^25^, ^15^.

In present study, 1135 cases of CL were discovered from five tehsils of Dera Ismail Khan district, KP. In comparison to the other 4 tehsils, tehsil D.I.K (62.7%) had the highest percentage of cases, followed by tehsil Paharpur (9.6%), Paroa (7%), Kulachi (5.8%), and Daraban (1.9%), which had the lowest percentage of cases. CL is widespread in several regions of Pakistan with various meteorological and geographic characteristics. While few instances have also been recorded from Sindh and Punjab, it is largely endemic in KP 26, 27. Demographic, socioeconomic, movement from rural to urban areas, religious, cultural, disturbance in climate (flood, drought), environmental factors, and/or insecurity (war/civil convict), people increasingly leaving their hamlets and heading to cities, poor hamlets are major risk factors that represent acute CL^28^. The other major source of risk is cross-border travel. North-western Pakistan experienced an epidemic that started in an Afghan emigrant camp a few years ago. It is thought that infectious migrant carriers of Kabul were the source of outbreak^25^. Another reason for the most cases originated from the tehsil Dera Ismail Khan was because of DHQ center which is easily accessible for the diagnosis and treatment of CL to the residents of D.I.K tehsil and nearby tehsils, also patient coming from other localities from various KP districts and a small number of cases coming from Afghan localities.

Most people who are sick were between the ages of 1 and 20, coincides with the previous studies 29, 30. Children were at a high risk of vector bites when playing outside in the shade of trees or close to moist ground surfaces where sandflies breed. Additionally, their immature and compromised immune systems increased their vulnerability to infection^31^, ^32^. According to current findings, out of a total of 1135 cases, the majority (62% of cases were involving men, like different districts of KP province^15^, ^21^. This could be due to societal norms in Pakistan, where males were more vulnerable to sandfly bites because they went to labour with their faces, shoulders, and hands exposed, whereas females cover themselves and stay indoors most of the time^29^.

The present results showed 23.9% people had multiple lesions and 76.1% people had a single lesion, consistent with previous studies^20^,^33^. Variations in shelter, circumstances, food habits, occupational activity, and host immunity to the infection can all lead to changes in the number of lesions^34^. Similarly, previous study recorded that most of the injuries occurred on exposed body regions, including the face, upper and lower body parts 20.The patient visited the hospital for the first time on average 12 to 14 weeks after the lesion first developed, all patients had early and late lesions, consistent with the previous study^15^. However, a study recorded that most CL patients had lesions that last between two and six months in length^35^. Similarly, studied recorded most of the lesions were treated within a year 36.

A choropleth map was used to display the spatial pattern of CL patients. The continuous colour represented the variation in patient abundance across tehsils. DIK had more patients reported, whereas Kulachi and Daraban had fewer. A visual representation of case distribution would make it easier for public health organisations to evaluate the severity of the infectious disease at the regional level 37. The extracted DEM map revealed the incidence of CL has been decreased with high altitudes and found highest in union council of Zaffar Abad with low elevation. The results were consistent with previous findings in different districts of KP province^15^, ^21^ who recorded vertical dissemination of CL would be restricted by the activities of sandflies, which prefer warm weather at low altitudes.

The IDW analysis justified the threats of CL infection in the areas located closer to the highest CL presenting villages. This could be explained based on the supposition that sandflies are weak flyers, the likelihood of contracting the disease decreased with increasing distance from an existing case. The CL occurrence of nearby locations was estimated using spatial interpolation because nearby locations shared similar characteristics such as land use, inhabitants' lifestyles, and sandfly breeding sources. The interpolation might serve as a reference for public health fields regarding disease prevention 35. The risk of CL has been further determined via cluster and outlier analysis. Apparent clusters were found in areas surrounding the current CL-prevalent tehsils, including D.I. K and Paharpur. When evaluating the effects of environmental factors on CL distribution, we found no evidence that a high incidence of CL was significantly correlated with high temperatures, high relative humidity, or rising annual precipitation. This could be explained by the fact that the time between the onset of the CL lesion and the diagnosis, on average the patient went to the hospital for the first time 12–14 weeks after the lesion initially appeared^15^. The emergence of CL transmission in regions with the lowest temperatures may be caused by movement of non-infected residents to endemic regions 38,39. Previous research has found that warm weather and precipitation help to spread CL because the development of both sandflies and Leishmania accelerates at high temperatures, and precipitation creates more suitable breeding sites for sandflies^40^.

## Conclusion

The research provided an overview of CL in the D.I. Khan district of Khyber Pakhtunkhwa, Pakistan. Males and those under the age of 20 made up the majority of the patient population. Infection was most common during the winter. The disease's spatial patterns were shown using GIS tools, and significant clusters have been identified in D.I.K and Paharpur tehsil. Identifying risk areas would assist public health sectors in forecasting future epidemics and launching appropriate interventions.

## Data Availability

The datasets supporting the conclusion of this article are included within the article.
